# Molecular Identification of Meningitis/Septicemia Due to *Streptococcus* spp. in Greece (2015–2024)

**DOI:** 10.3390/diagnostics15131632

**Published:** 2025-06-26

**Authors:** Constantinos Karamalis, Athanasia Xirogianni, Stelmos Simantirakis, Marina Delegkou, Anastasia Papandreou, Georgina Tzanakaki

**Affiliations:** National Meningitis Reference Laboratory, Department of Public Health Policy, School of Public Health, University of West Attica, 1152 Athens, Greece; kkaramalis@uniwa.gr (C.K.); ssimantirakis@uniwa.gr (S.S.); mdelegkou@uniwa.gr (M.D.); npapandreou@uniwa.gr (A.P.); gtzanakaki@uniwa.gr (G.T.)

**Keywords:** streptococcal meningitis, *Streptococcus* spp., molecular identification, PCR, sequencing, Greece, epidemiology

## Abstract

**Background/Objectives**: Meningitis due to the species *Streptococcus* is a severe central nervous system infection caused by various microorganisms belonging to *Streptococcus* spp. Its accurate identification is critical for effective clinical management. This study aimed to identify *Streptococcus* spp. causing meningitis in Greece over a nine-year period using PCR and sequencing-based methods. **Methods**: A total of 189 clinical samples, collected between 2015 and 2024 from patients suffering from meningitis and/or septicemia, were analyzed by the use of a combination of multiplex polymerase chain reaction (PCR) assays and *tuf* gene sequencing for further species identification. **Results**: Sample analysis identified 70 samples as *S. pyogenes* (18.52%) (GAS) and *S. agalactiae* (18.52%) (GBS), while 119 (62.96%) were recorded as non-typable *Streptococcus* spp. Further analysis using sequencing methods revealed that the most frequent *Streptococcus* spp. belonged to the mitis group (42.86%) and the pyogenic group (20.17%). A higher prevalence was observed in children aged 0–14 years old and adults over 50 years old. **Conclusions**: This study highlights the use of molecular diagnostics in identifying other *Streptococcus* spp., providing insights into age-related susceptibility and epidemiological trends. Future studies are needed to explore the pathogenic role of the identified *Lactococcus* spp.

## 1. Introduction

Meningitis is a severe central nervous system (CNS) infection, caused mainly by *Neisseria meningitidis*, *Streptococcus pneumoniae* and *Haemophilus influenzae* type b [1,2], and to a lesser extent by *Streptococcus pyogenes* (GAS), *Streptococcus agalactiae* (GBS), *Listeria monocytogenes* and *Escherichia coli* [3,4]. However, there are other *Streptococcus* spp. causing meningitis. According to the literature, they belong to various species other than *S. pneumoniae*, *S. pyogenes* (GAS) and *S. agalactiae* (GBS) [5]. Those *Streptococcus* spp. that are described as α- hemolytic belong to the Viridans Streptococcus Group; for instance, *Streptococcus mitis*, which colonizes the oral cavity [6,7]. Moreover, there are other streptococcal groups (GCS and GGS) also causing meningitis or septicemia; for example, *Streptococcus constellatus* subs. *constellatus* and *Streptococcus dysgalactiae*, as part of the human upper respiratory tract flora and that often colonize the skin, oral cavity and the female genitals [8,9,10,11].

The classification of *Streptococcus* spp. is based on some of their specific characteristics, such as their hemolytic reactions, as well as other biochemical traits [7]. Based on their classification, streptococci are identified as alpha (α) hemolytic (causing the incomplete rupturing of red blood cells), beta (β) hemolytic (causing the complete lysis of erythrocytes) and gamma (γ) hemolytic (for which no lysis is observed).

Further typing was developed in 1933 by Lancefield [12], and the utilization of the Lancefield group antigens along with the 10 other phenotypic characteristics by Sherman (1937) [13] resulted in an early identification of the genus into the following four main groups: “pyogenic”, “viridans”, “lactic” and “enterococci”. The pyogenic β-hemolytic group carrying different Lancefield group antigens included *S. pyogenes* (GAS), *S. agalactiae* (GBS), Group C (GCS), Group D (GDS), Group E (GES), Group F (GFS) and Group G (GGS [7,12,14].

Although the Lancefield serotyping was initially successful in terms of species grouping, it was evident that streptococci, physiologically quite heterogeneous, could possess a common group antigen, a fact that caused confusion in early streptococcal taxonomy. Hence, by the use of molecular methods—such as initially DNA-DNA and DNA-rRNA [15], then hybridization to 16S rRNA [16] and finally to genus-specific PCR genes such as the *16S rRNA* gene and the *Tuf* (elongation factor Tu) gene [17]—was generally successful. Based on the sequence analysis of the *16S rRNA* gene, the streptococci genus was found to include approximately 79 different species, which are divided into six species groups: the anginosus group, the mitis group, the mutans group, the pyogenic group and the salivarius group [7,18].

Although conventional identification based on biochemical characteristics remains the gold standard for microbiology laboratories, those methods are not always reliable in terms of distinguishing among the different *Streptococcus* spp. groups due to gene transfer [19]. Furthermore, the fact that these methods are time-consuming and sometimes produce false negative results, the development of molecular techniques is essential for further typing [20,21], especially for culture-negative PCR-positive samples.

This study aims to identify *Streptococcus* spp. causing meningitis and/or septicemia in Greece during a nine-year period (2015–2024) by the use of molecular tools to directly culture negative PCR-positive clinical samples in order to generate further information on streptococcal meningitis and/or septicemia.

## 2. Materials and Methods

### 2.1. Source of Specimens and Data Collection

Between January 2015 and December 2024, a total of 189 culture-negative clinical samples PCR-positive for *Streptococcus* spp. (with the exclusion of *Streptococcus pneumoniae*) obtained from 189 patients with meningitis and/or septicemia were sent to the National Meningitis Reference Laboratory from hospitals all over the country. Among those, meningitis was present in 119 patients, while septicemia was present in 70 patients. The samples of CSF (*n* = 119) and blood (*n* = 70), respectively, were examined, including 10 cases in which meningitis and septicemia were both present.

**Figure 1:**
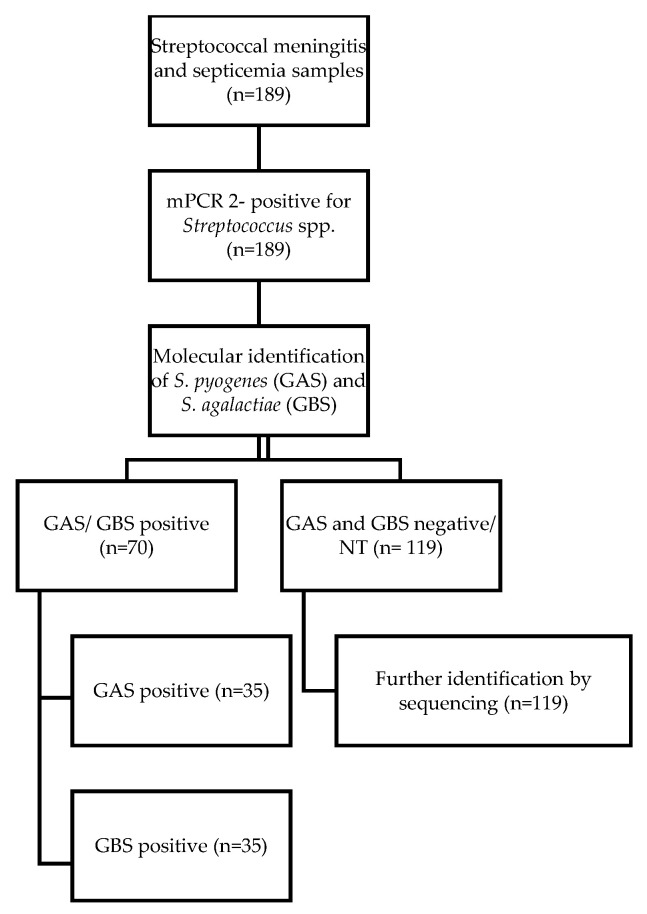
Flowchart for sample identification included in the study.

Of the 189 samples, *S. pyogenes* and *S. agalactiae* were identified in 37.04% (70/189), while 119 clinical samples (62.96%; 119/189) were further identified by the application of the amplification and sequencing of the *tuf* gene (Figure 1).

### 2.2. Streptococcus spp. Identification

A multiplex PCR was deployed for the simultaneous identification of *Streptococcus* spp., as described previously [22]. Further typing was carried out for the identification of GAS and GBS by the use of a single PCR assay (Figure 1).

#### Identification of *S. pyogenes* (GAS) and *S. agalactiae* (GBS)

Amplification reactions contained 0.4 μmol/L of the SpyF/SpyR (GAS) and CFBA/CFBS (GBS) primers (Eurofins Genomics AT GmbH, Vienna, Austria) (Table 1), respectively, 1 X KAPA 2G Fast Multiplex Mix (Sigma-Aldrich Chemie GmbH, Schnelldorf, Germany) and a 3 μL DNA template in a total volume of 20 μL. The polymerase chain reaction conditions were 95 °C for 3 min; 95 °C for 15 s; 58 °C for 15 s; and 72 °C for 30 s for the first 15 cycles. This was followed by 18 cycles at 95 °C for 15 s, 57 °C for 15 s, 72 °C for 30 s and a final step of 72 °C for 1 min (VWR Doppio Gradient 2 × 48 well Thermocycler, VWR International GmbH, Darmstadt, Germany).

A total of 20 μL of PCR amplicons was visualized under ultraviolet fluorescent light following electrophoresis in 2% (*w*/*v*) agarose gel stained with 3 μL pf GelRed loading buffer (6× Gel loading dye, Biotium, Fremont, CA, USA). Positive controls from standard strains of *Streptococcus pyogenes* and *Streptococcus agalactiae* (5 ng of each DNA), as well as negative controls, were included in each assay (Figure 1).

### 2.3. Further Streptococcus spp. Identification

For the 119 samples (62.96%) that were not identified either as GAS or GBS (Figure 1), further molecular identification was deployed using a PCR sequencing-based method, aiming to enhance the *tuf* gene and encoding the elongation factor Tu included in the bacterial genome [17].

#### *Tuf* Gene Amplification

Each PCR contained 0.5 μmol/L of Str1 and Str2 primers (Eurofins Genomics AT GmbH, Vienna, Austria) (Table 1), 0.8 mmol/L dNTPs (New England Biolabs, Ipswich, MA, USA), 0.24 U/reaction Phusion^®^-High Fidelity DNA Polymerase (New England Biolabs Ipswich, MA, USA), 1.2 X GC reaction buffer (New England Biolabs, Ipswich, MA, USA) and a 2 μL DNA template in a total volume 20 μL. The polymerase chain reaction conditions were 98 °C for 30 s; 98 °C for 5 s; 65 °C for 12 s; and 72 °C for 15 s for the first 11 cycles. This was followed by 27 cycles at 98 °C for 5 s, 63 °C for 12 s, 72 °C for 15 s, and a final step of 72 °C for 1 min (VWR Doppio Gradient 2 × 48 well Thermocycler, VWR International GmbH, Darmstadt, Germany).

A total of 5 μL of PCR amplicons was visualized under ultraviolet fluorescence following electrophoresis in 2% (*w*/*v*) agarose gel stained with 1 μL of GelRed loading buffer (6× Gel loading dye, Biotium, Fremont, CA, USA). Positive controls from standard strains of *Streptococcus pyogenes* (5 ng of each DNA), as well as negative controls, were included in each assay. Primers for the *Streptococcus* spp. *tuf* gene (Str1/2) were based on those described previously [17] with the resulting amplicon sizes of 198 bp. DNA products were purified and sequenced.

### 2.4. PCR Purification

PCR amplicons were purified according to the PCR-clean-up protocol, NucleoSpin^®^ Gel and PCR Clean-up kit (Macherey–Nagel, Düren, Germany) in a 25 μL final elution volume. The purification yield was tested by electrophorizing 5 μL of the purified product, which was stained with 1 μL of GelRed loading buffer (6× Gel loading dye, Biotium, Fremont, CA, USA) in 2.0% (*w*/*v*) agarose gel (Nippon Genetics, Tokyo, Japan) and visualized under ultraviolet fluorescent light. The purified products were sent for sequencing.

### 2.5. Sequencing Analysis

The results of the sequencing analysis (chromatograms) were analyzed and edited by Chromas software (http://technelysium.com.au/wp/chromas/, accessed on 20 December 2024, version 2.6.6 (Technelysium Pty Ltd., South Brisbane, Australia, downloaded for free). The nucleotide sequences derived from the two DNA chains were compared to each other with ClustalW (https://www.genome.jp/tools-bin/clustalw, accessed on 20 December 2024, Bioinformatics tools provided by GenomeNet, Kyoto University Bioinformatics Center, Kyoto, Japan, free online software). The nucleotide sequences were imported into BLAST + 2.15.0 (https://blast.ncbi.nlm.nih.gov/Blast.cgi?PROGRAM=blastn&PAGE_TYPE=BlastSearch&LINK_LOC=blasthome, accessed on 20 December 2024) and the results were automatically assigned.

## 3. Results

### 3.1. PCR Amplification

**Figure 2:**
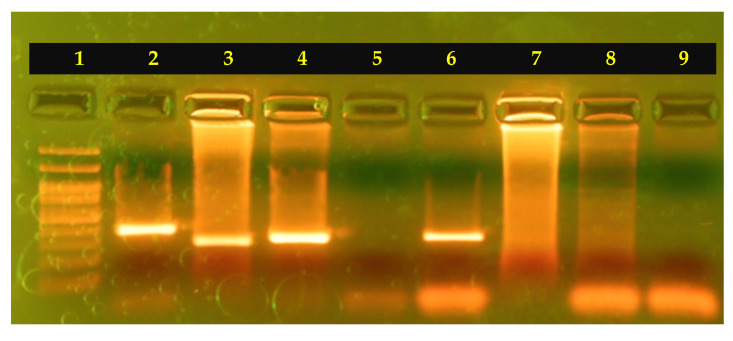
GAS and GBS molecular identification by PCR assay of lanes: (1) 100 bp ladder; (2,3) positive controls (5 ng of each DNA)—GAS (MW: 341 bp) and GBS (MW: 259 bp); (4,6–8) clinical samples; and (5,9) negative controls.

The amplification of the target genes formed products of 341 bp (GAS) and 259 bp (GBS), respectively (Figure 2). The GAS and GBS identification was positive for 70 of 189 samples (37.04%). Specifically, 35 of the 70 samples were identified as GAS and 35 of the samples were identified as GBS, respectively.

**Figure 3:**
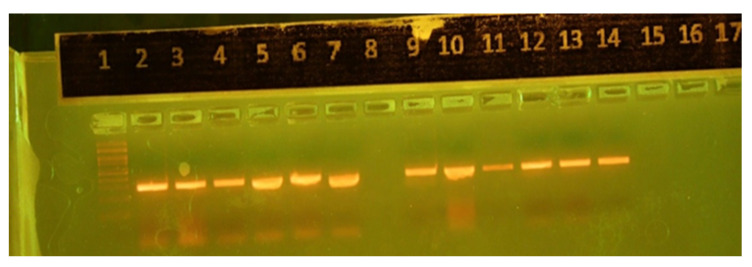
*Tuf* gene amplification assay. Lanes: (1) 100 bp ladder; (2) positive control (MW: 198 bp); (8) negative control; and (3–7,9–14) clinical samples.

The PCR amplicons of the *tuf* gene PCR assay were 198 bp. Moreover, this technique provided positive results for most of the cases (116 (97.5%) out of 119 clinical samples) (Figure 3).

### 3.2. Sequencing Results

Sequencing analysis revealed that the majority of the genus *Streptococcus* spp. belonged to the Viridans Streptococci Group (VS), Bovis, Pyogenic Group and *Lactococcus* spp. According to the sequencing results, the most prevalent group was the mitis group (43%; 51/119). Among this group, *S. mitis* was successfully identified in 30 samples (59%; 30/51), followed by *S. oralis* in 21 samples (41%; 21/51). Furthermore, among the 17 streptococci belonging to the Anginosus group, *S. anginosus* and *S. intermidius* were identified in seven samples (41%; 7/17) each, respectively, while *S. constellatus* was identified in three samples (3/17; 18%).

**Figure 4:**
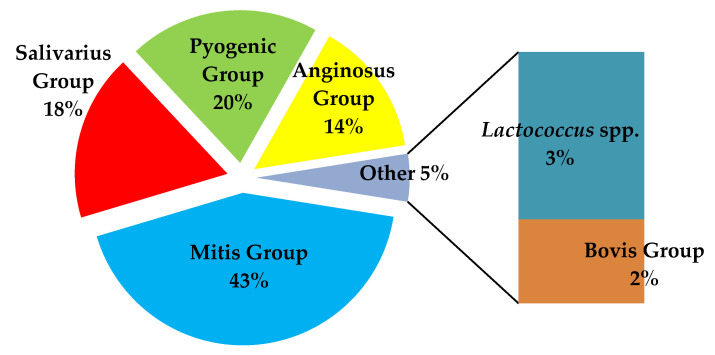
Sequencing analysis of *tuf* gene directly in clinical samples.

Among the pyogenic group, *S. dysgalactiae* was identified in all 24 samples (20.1%; 24/119). In addition, among the salivarius group, *S. salivarius* was identified in all 21 samples (18%; 21/119). Finally, among the two positive samples belonging to Bovis Group, *S. bovis* was identified. Further, *Lactococcus* spp. was identified in all 3 samples (3%) (Figure 4).

**Figure 5:**
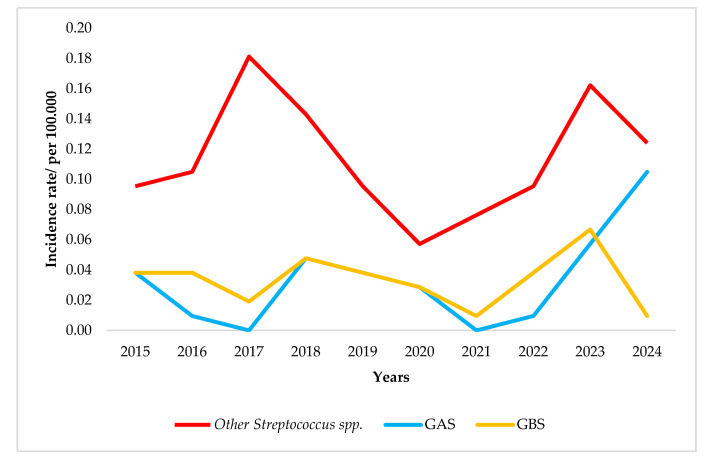
Incidence rate of streptococcal meningitis/septicemia in Greece (2015–2024).

Further analysis of the sequencing results over time revealed that the mitis group was predominant throughout the target years, followed by the salivarius group and the pyogenic group. Specifically, an increase in the mitis group, although observed during the pre-pandemic period (2017–2018), with the exception of the COVID-19 period (2020–2021), was also observed during the post-COVID-19 era (2022–2024), almost reaching pre-pandemic levels (Figure 5).

**Figure 6:**
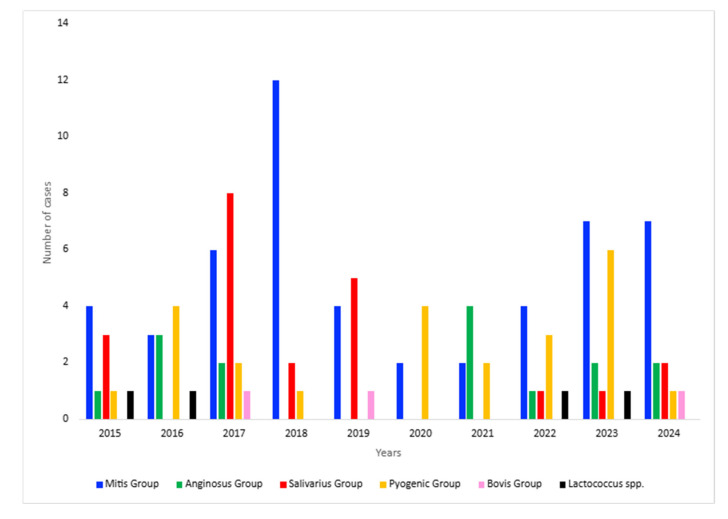
Annual cases of meningitis and septicemia from other streptococcal species (2015–2024).

Regarding the number of meningitis and/or septicemia cases caused by the pyogenic group and, in particular, *S. dysgalactiae*, an increase was observed in 2022–2023, while a decrease was observed in 2024 (Figure 6).

### 3.3. Contribution to Laboratory Surveillance

In general, the annual average incidence rate (IR) for meningitis and septicemia due to *S. pyogenes* (GAS) was 0.03/100,000. However, the incidence rate varied throughout the study period. Specifically, the pre-pandemic period was observed in 2018 (0.04/100,000). However, although during the pandemic period there were no GAS cases, a gradual increase was observed during the post-pandemic period (IRs of 0.02, 0.06 and 0.09 per 100,000 for 2022, 2023 and 2024, respectively) (Figure 5).

On the other hand, meningitis and septicemia caused by *S. agalactiae* (GBS) presented a different pattern in the annual incidence rate. Specifically, the incidence rate seemed to adhere to an average rate of 0.03/100,000 throughout the study years, while a decrease attributed to COVID-19 pandemic measures was observed in 2021. Although a peak was observed during 2021–2023 with an average rate of 0.02/100,000 population, the increasing trend was not observed for 2024 (Figure 5).

With regard to the remaining *Streptococcus* spp., identified neither as GAS nor GBS, an increase in the incidence rate was observed in 2017 (0.18/100,000) while a dramatic reduction was observed during the following years (2018–2020, average IR 0.11/100,000). However, an increase in the incidence rate was observed during the post-COVID-19 period (2021–2023)—with an average rate of 0.16/100,000—although a slight decline (0.12/100,000) was observed during 2024 (Figure 5).

### 3.4. Age Distribution

**Figure 7:**
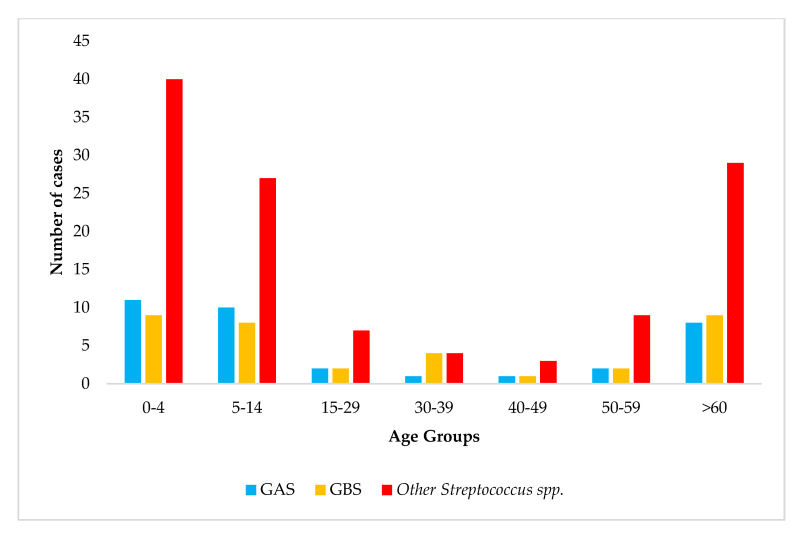
Age distribution of meningitis and septicemia due to streptococci in Greece (2015–2024).

With regard to age, for most of the meningitis and septicemia cases due to *Streptococcus* spp., the highly affected age group was 0–4 years old (31.8%; 60/189) followed by the age groups > 60 years old (24.8%; 46/189) and 5–14 years old (23.8%; 45/189). In general, *Streptococcus* spp. other than GAS and GBS were responsible for the highest percentage of meningitis/septicemia cases in this study (Figure 7).

**Figure 8:**
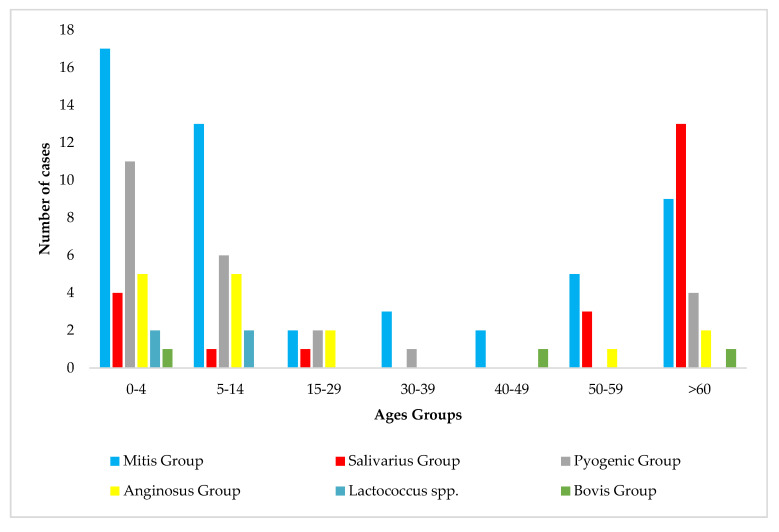
Age distribution in relation to *Streptococcus* spp. identification.

Analysis of the identified streptococci in relation to age revealed that the most predominant species was the mitis group streptococci, especially in children aged 0–4 years old (33%; 17/51) and 5–14 years old, as well as in adults >60 years of age (30%; 9/30) (Figure 8).

Salivarius group streptococci were found mainly in adults over 60 years of age (43%; 13/30) (Figure 8).

The pyogenic group of *Streptococcus* spp. was found in children 0–4 (21%; 11/51) and 5–14 years old (12%; 6/51), as well as in adults over 60 years old (13%; 4/30) (Figure 8).

Furthermore, *Lactococcus* spp. were found in children aged 0–4 (two cases) and 5–14 years old (two cases), respectively (Figure 8).

## 4. Discussion

The identification of *Streptococcus* spp. is important as it provides a great and vital amount of information on their biology and pathogenicity. Although conventional techniques are commonly used for the identification of bacteria (e.g., cultures and biochemical tests), the development and application of molecular assays resolves important issues in laboratory practice, especially when it comes to the surveillance of microorganisms [25].

In the case of *Streptococcus* spp., identification by culture combined with biochemical assays is not always possible in cases such as mitis group Streptococci (the main species of which are *Streptococcus pneumoniae*, *Streptococcus mitis* and *Streptococcus oralis*), for which some biochemical characteristics are presented in more than one species. For instance, while *S. pneumoniae* colonies are usually mucous, several cases lack this characteristic. Furthermore, as optochin testing was one of the main characteristics tests for *S. pneumoniae*, recent studies have indicated that streptococci belonging to the *S. mitis* species also present the same sensitivity to optochin [20]. Hence, molecular identification methods have an advantage in terms of further identification over the conventional ones as they are more specific and sensitive and less time-consuming [26].

Specifically, the application of the combined assays used in the present study successfully identified the majority of the genus *Streptococcus* spp. belonging to the Viridans Streptococci Group (VS), the mitis, salivarius and anginosus groups, as well as the bovis and pyogenic groups [18]. Further, the *tuf* gene sequencing assay also identified *Lactococcus* spp., which is in agreement with previous studies [17].

With regard to age, mitis group streptococci (*S. mitis*/*pseudopneumoniae*, *S. oralis*) were identified in patients 0–4 years old and in adults > 50 years old, which is in agreement with clinical case studies indicating that meningitis/septicemia from *Streptococcus mitis/pseudopneumoniae* and *Streptococcus oralis* occur mainly in children, as well as in older adults, due to underlying diseases and weak immune systems [6,27,28,29]. *S. salivarius* is one of the first microorganisms to colonize the mucosa, in the very first days after birth. Although it is not pathogenic, it can cause infections, leading to sepsis and meningitis [30], as well as in cases associated with surgery; e.g., epidural anesthesia or myelography [30,31]. According to our results, a significant percentage of *S. salivarius* was found in patients of >50 years of age, which is in agreement with previous studies [31,32], as well as in children 0–4 years of age.

The anginosus group (SAG) is usually found in the normal mucosal flora, colonizing both the urinary tract and the female genitals [33,34]. However, they could be potentially pathogenic in aseptic environments, such as CSF, and may cause severe infections. The aforementioned streptococcal group was identified in a low number of cases (14%) in all age groups, which is in agreement with previous studies indicating that infections caused by *S. anginosus* do not follow any specific age pattern [35,36].

*Streptococcus dysgalactiae* is a member of the pyogenic group; infections are associated with skin infections, which cause high rates of bacteremia, resulting in meningitis mainly in children [37]. This is in agreement with our findings as *Streptococcus dysgalactiae* was identified in most of the samples (20%) taken from children 0–15 years old.

*Lactococcus* spp. was identified in four samples (3%; 4/119) from the age groups 0–2 years old and 5–14 years old, respectively. This is an interesting observation indicating that the transmission of *Lactococcus* spp. might occur during breastfeeding as—according to previous studies—it has been found to be present in breast milk, among other bacteria [38]. Naturally, bovines are the natural reservoirs of *Lactococcus* spp., which can infect humans through the consumption of contaminated raw milk or fermented dairy products. Upon ingestion, *Lactococcus lactis* may colonize the human gastrointestinal tract and may potentially cause infections by invading sterile body sites [39].

The proposed methodology has advantages over Matrix-Assisted Laser Desorption/Ionization Time of Flight (MALDI-TOF), as MALDITOF is highly dependent on both the quality and thoroughness of the database [40]. Furthermore, as a low bacterial load in clinical samples such as CSF or blood often occurs, this may require additional steps such as sample purification, which is complex and time-consuming [41]. Furthermore, using MALDI-TOF MS to identify closely related microorganisms could be difficult for genetically related species such as *Streptococcus* spp. with similar protein profiles [42]. In such cases, the development of molecular methods, such as the PCR sequencing-based method, provides more reliable results.

PCR sequencing-based methods are fast, reliable, cost-effective, and suitable for small laboratories for monitoring streptococcal infections such as meningitis and septicemia.

## 5. Conclusions

In conclusion, no previous studies have focused on the molecular identification of meningitis and septicemia/bacteremia caused by *Streptococcus* spp. in the context of epidemiological monitoring. The existing literature primarily consists of individual case reports, which lack further specific identification and comprehensive data for epidemiological analysis. This study, therefore, presents a significant advance in the field, providing essential molecular insights into the prevalence, identification, and impact on public health of *Streptococcus* spp. related to severe infections.

The development of a protocol that can be applied directly to clinical samples initially contributes to the reduction in the turnaround time required for identification, without relying on culture-based methods. This advantage ensures the production of reliable and reproductive results, while minimizing the risk of contamination as the sample undergoes minimal processing before the assay’s application. Moreover, the application of this protocol revealed new data concerning meningitis and septicemia/bacteremia due to *Streptococcus* spp. other than *S. pneumoniae*, GAS and GBS. Finally, the implementation of the present molecular technique has significantly enhanced the laboratory-based surveillance of streptococcal meningitis.

## Figures and Tables

**Table 1:** Oligonucleotide sequencing and PCR primers used for amplification of Spy1258 (GAS), cfb (GBS) and *tuf* (other *Streptococcus* spp.) genes.

Primers	Encoding Gene	Sequence 5′ → 3′	Product	Reference
SpyF SpyR	*spy1258*	ACTCTGGATGATTTGTACCG TCAGTGGTTTCTTGATAGCC	314 bp	[23]
CFBS CFBA	*cfb*	ATGATGTATCTATCTGGAACTCT CGCAAT GAAGTCTTTAATTTTTC	259 bp	[24]
Str1 Str2	*tuf*	GTACAGTTGCTCAGGACGTATC ACGTTCGATTTCATCACGTTG	198 bp	[17]

## Data Availability

The original contributions presented in this study are included in the article. Further inquiries can be directed to the corresponding author.
